# A phase II study of talimogene laherparepvec for patients with inoperable locoregional recurrence of breast cancer

**DOI:** 10.1038/s41598-021-01473-2

**Published:** 2021-11-15

**Authors:** Megumi Kai, Angela N. Marx, Diane D. Liu, Yu Shen, Hui Gao, James M. Reuben, Gary Whitman, Savitri Krishnamurthy, Merrick I. Ross, Jennifer K. Litton, Bora Lim, Nuhad Ibrahim, Takahiro Kogawa, Naoto T. Ueno

**Affiliations:** 1 Department of Breast Medical Oncology, The University of Texas MD Anderson Cancer Center, 1515 Holcombe Blvd., Unit 1354, Houston, TX 77030 USA; 2 Morgan Welch Inflammatory Breast Cancer Research Program and Clinic, The University of Texas MD Anderson Cancer Center, Houston, TX USA; 3 Department of Biostatistics, The University of Texas MD Anderson Cancer Center, 1515 Holcombe Blvd., Unit 1354, Houston, TX 77030 USA; 4 Department of Hematopathology Research, The University of Texas MD Anderson Cancer Center, 1515 Holcombe Blvd., Unit 1354, Houston, TX 77030 USA; 5 Department of Breast Imaging, The University of Texas MD Anderson Cancer Center, 1515 Holcombe Blvd., Unit 1354, Houston, TX 77030 USA; 6 Department of Anatomical Pathology, The University of Texas MD Anderson Cancer Center, 1515 Holcombe Blvd., Unit 1354, Houston, TX 77030 USA; 7 Department of Surgical Oncology, The University of Texas MD Anderson Cancer Center, 1515 Holcombe Blvd., Unit 1354, Houston, TX 77030 USA; 8 Present Address: Department of Oncology/Medicine, Baylor College of Medicine, 7200 Cambridge St., Houston, TX 77030 USA

**Keywords:** Medical research, Oncology, Cancer, Breast cancer, Cancer therapy, Cancer

## Abstract

Talimogene laherparepvec (T-VEC) is an immunotherapy that generates local tumor lysis and systemic antitumor immune response. We studied the efficacy of intratumoral administration of T-VEC as monotherapy for inoperable locoregional recurrence of breast cancer. T-VEC was injected intratumorally at 10^6^ PFU/mL on day 1 (cycle 1), 10^8^ PFU/mL on day 22 (cycle 2), and 10^8^ PFU/mL every 2 weeks thereafter (cycles ≥ 3). Nine patients were enrolled, 6 with only locoregional disease and 3 with both locoregional and distant disease. No patient completed the planned 10 cycles or achieved complete or partial response. The median number of cycles administered was 4 (range, 3–8). Seven patients withdrew prematurely because of uncontrolled disease progression, 1 withdrew after cycle 3 because of fatigue, and 1 withdrew after cycle 4 for reasons unrelated to study treatment. Median progression-free survival and overall survival were 77 days (95% CI, 63–NA) and 361 days (95% CI, 240–NA). Two patients received 8 cycles with clinically stable disease as the best response. The most common grade 2 or higher adverse event was injection site reaction (n = 7, 78%). Future studies could examine whether combining intratumoral T-VEC with concurrent systemic therapy produces better outcomes.

## Introduction

Following mastectomy or breast-conserving surgery, the 10-year incidence of locoregional breast cancer recurrence is about 12%^1,2^. Locoregional recurrence can result in substantial morbidity, and it also frequently leads to systemic metastases^1,3,4^. If the locoregional recurrence is locally limited, complete surgical resection and radiotherapy followed by systemic therapy can be considered depending on prior therapy^5–8^. However, if the locoregional recurrence manifests with diffuse skin disease, invasion into the surrounding structures, or advanced lymphadenopathy, disease is often inoperable. While systemic therapy is primarily indicated for patients with inoperable locoregionally recurrent breast cancer^8,9^, the treatment with multiple lines of systemic therapy increases the resistance of the disease, and the prognosis is typically poor. Therefore, there remains a strong need for new treatment modalities that will control locoregionally recurrent breast cancer effectively.

Talimogene laherparepvec (T-VEC) is an attenuated herpes simplex virus type 1 (HSV-1) that can replicate only in tumor cells^10–14^. T-VEC has been genetically modified to express GM-CSF to enhance the immune response by activating dendritic cells and stimulating T cells^15,16^. T-VEC is administered by direct injection into cutaneous or subcutaneous disease deposits and generates a systemic antitumor immune response. In a phase I trial in 2006, T-VEC was administered intratumorally in 30 patients with cancer in whom prior therapy had failed (14 with breast cancer, 9 with melanoma, 2 with colorectal cancer, and 5 with head and neck cancer)^17^. In this study, patients received either a single dose or 3 doses of T-VEC and were observed for 6 weeks after their last injection. T-VEC was well tolerated, and local inflammation, erythema, and febrile responses were the main side effects. Among the 14 breast cancer patients, 9 received a single dose, and 5 received 3 doses. Four of the breast cancer patients had local disease stabilization, but there were still issues controlling disease progression at non-injected lesions.

The purpose of the study we describe here was to determine the local and systemic antitumor efficacy of T-VEC as monotherapy for inoperable locoregional recurrence of breast cancer with or without distant metastases.

## Patient and methods

This was a single-center, open-label, single-arm phase II study (ClinicalTrials.gov Identifier: NCT02658812). The protocol was approved by the institutional review board at The University of Texas MD Anderson Cancer Center. The study was conducted in accordance with the Declaration of Helsinki and adhered to Good Clinical Practice guidelines. All enrolled patients provided written informed consent.

### Eligibility

To be eligible, patients had to have histologic confirmation of breast cancer recurrence with chest wall/cutaneous, subcutaneous, or nodal tumors with or without distant metastases. Patients also had to have at least 1 injectable lesion ≥ 5 mm in longest diameter or multiple injectable lesions with longest diameters totaling ≥ 5 mm. Furthermore, patients had to have received at least 1 systemic therapy regimen for the recurrent disease. An ECOG performance status of 0–1 and adequate organ function were required. Exclusion criteria included disease amenable to surgery with curative intent; metastatic sites that required urgent systemic chemotherapy; known active central nervous system metastases; > 3 lesions per organ in the case of visceral metastases other than lung or lymph node metastases; a history of prior complications from HSV-1 infection; and active autoimmune disease requiring systemic treatment.

### Treatment plan

The trial was planned to conduct using a two-stage design, and the overall response rate was estimated accordingly. It was assumed that the T-VEC single agent would have a response rate of 20%. A 5% or lower response rate will be considered treatment failure, and the regimen will be rejected under this circumstance. 13 patients were planned to enroll in the first stage. If no patients achieve an overall response to the treatment, the trial will be stopped, and the regimen will be declared ineffective. If there are 1 or more patients achieve an overall response, 22 more patients will be enrolled to the study to reach a total of 35 treated patients.

Before administration of T-VEC, all patients underwent imaging and had baseline medical photographs taken to serve as references. T-VEC was injected into the visible sites of locally recurrent breast cancer or skin metastases on day 1 (cycle 1), day 22 (cycle 2), and every 2 weeks thereafter (cycles ≥ 3) until disease progression. The T-VEC concentration in the initial cycle was 10^6^ PFU/mL, irrespective of HSV-1 serology status. The T-VEC concentration in the second and subsequent cycles was 10^8^ PFU/mL. The total dose of T-VEC injected varied according to tumor size but did not exceed 4.0 mL in any injection cycle^18^. Medical photographs of local lesions were taken before each cycle to record the extent of the disease. Imaging was planned for every 5 cycles until disease progression.

T-VEC was to be permanently discontinued if patients required another anticancer therapeutic agent for any reason, if therapy was delayed > 6 weeks because of a grade 2 or higher immune-mediated adverse event (AE)/allergic reaction, or if therapy was delayed > 4 weeks because of any other T-VEC-related grade 3 or higher toxicities. We designed the study to administer T-VEC for at least 10 cycles (approximately 5 months) unless uncontrolled disease progression was observed. Beyond 10 cycles, progressive disease will be measured based on RECIST ver1.1. Uncontrolled disease progression was defined as the rapid growth of multiple measurable or nonmeasurable new lesions or increase in the sum of the longest diameters of existing targeted lesions > 40% from the baseline. We chose 10 cycles of treatment because a previous study of intralesional T-VEC injections for advanced melanoma showed that more than half of the patients experienced pseudo-progression (an increase in the size of lesions or appearance of new lesions) before they achieved a response, with median time to response of 4.1 months^13^.

### Toxicity assessment

AEs and laboratory results were graded using the Common Terminology Criteria for Adverse Events, version 4.0. Grade 2 or higher nonhematologic AEs and grade 3 or higher hematologic AEs were recorded upon observation by the investigator or report by the patient regardless of whether the AEs were attributed to the investigational product. Grade 2 or higher abnormal laboratory values were recorded as AEs as well. Dose-limiting toxicities were defined as the following AEs when they were at least possibly related to T-VEC: herpetic event requiring treatment, grade 3 or higher immune-mediated AE, grade 2 or higher allergic reaction, grade 3 nonhematologic toxicity lasting more than 3 days, grade 3 or higher nonhematologic abnormal laboratory value failing to respond to medical intervention or leading to hospitalization, grade 2 or higher febrile neutropenia, grade 4 thrombocytopenia, and grade 4 nonhematologic toxicity. In the case of dose-limiting toxicity, T-VEC administration was deferred until the toxicity had resolved to at least grade 1 or returned to baseline levels.

### Endpoints

The primary endpoint was the overall response rate, defined as the rate of patients who achieved a partial response or complete response as the best response for the measurable and nonmeasurable disease. The response was planned to evaluate using RECIST version 1.1 at the end of cycle 10 and onward. Secondary endpoints were rates of local overall response/disease control rate, progression-free survival, and overall survival.

### Correlative immune studies

Peripheral blood collected at baseline and before cycle 5 was analyzed to determine the subset of immune cells via multiparameter FACS (LSRII, BD Biosciences). We analyzed the percentage and absolute counts of total T cells (CD3^+^), helper T cells (CD4^+^), cytotoxic T cells (CD8^+^), B cells (CD19^+^), total NK cells (CD56^+^CD3^−^), NKT cells (CD56^+^CD3^+^), subset of NK cells (CD56^+^CD16^+^, CD56^+^CD16^−^, CD56^−^CD16^+^, CD56^+^CD57^+^), Treg cells, dendritic cells (DC), myeloid dendritic cells (mDC), and plasmacytoid dendritic cells (pDC). The paired *t* test was applied to compare between 2 time points.

### Statistical analysis

Progression-free survival and overall survival from the date of treatment initiation were estimated using the Kaplan–Meier method with 95% confidence intervals (CIs). Progression-free survival was defined as the time from treatment initiation until disease progression, and overall survival was defined as the time from treatment initiation until death.

## Results

### Patient characteristics

Ten patients enrolled in the study, and 9 patients were treated in the study. Of the 10 patients who enrolled, 2 patients withdrew consent before initiating treatment to pursue alternate treatment; of these 2, one patient re-enrolled and received treatment on this study. Thus, 9 patients were evaluable for toxicity and response. The median age was 49 years (range, 39–70 years), and all of the participants had noninflammatory breast cancer. Six patients were seropositive and 3 were seronegative for HSV-1 at enrollment. Three patients had distant metastases in addition to locoregional disease (Table 1).

**Table 1:** Demographic and clinical characteristics at baseline.

Characteristic	n (%)
Age, median (range), y	49 years (39–70)
**Sex**	
Female	9 (100)
Male	0
**Race**	
White	5 (56)
Black	3 (33)
Asian	1 (11)
**Ethnicity**	
Hispanic	0
Not Hispanic	9 (100)
**HSV-1 serology**	
Positive	6 (67)
Negative	3 (33)
**Type of breast cancer**	
IBC	0
Non-IBC	9 (100)
**Estrogen receptor status**	
Positive	2 (22)
Negative	7 (78)
**Progesterone receptor status**	
Positive	2 (22)
Negative	7 (78)
**HER2 status**	
Positive	1 (11)
Negative	8 (89)
**Distant metastases**	
No	6 (67)
Yes	3 (33)

*IBC* inflammatory breast cancer.

### Clinical response

**Figure 1:**
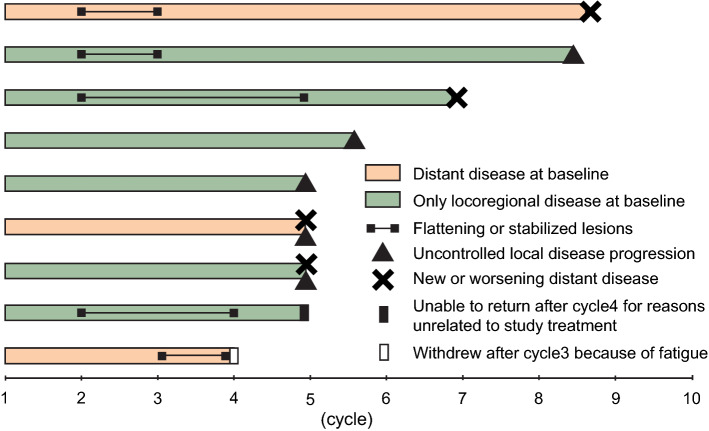
Swimmer plot showing clinical response for each patient. Each bar represents an individual patient.

There were no complete or partial responses. None of the patients were able to complete the scheduled 10 cycles (Fig. 1); therefore, the study was discontinued prematurely without completing enrollment for the first stage. Median progression-free survival and overall survival were 77 days (95% CI, 63–NA) and 361 days (95% CI, 240–NA). The median number of cycles received was 4 (range, 3–8). In 7 patients (78%), the study treatment was discontinued because of uncontrolled disease progression. Among those 7 patients, 3 patients had uncontrolled local disease, i.e., increase in tumor burden or appearance of new lesions; 2 patients developed new distant metastases; and 2 patients had both uncontrolled local disease and new or worsened distant metastases. Of the remaining 2 patients, 1 left the study after 4 cycles as she could not return to the clinic for reasons unrelated to study treatment, and 1 patient withdrew after 3 cycles because of fatigue (Fig. 1). These 2 patients had no images available to confirm the status of the local and the systemic disease at withdrawal.

**Figure 2:**
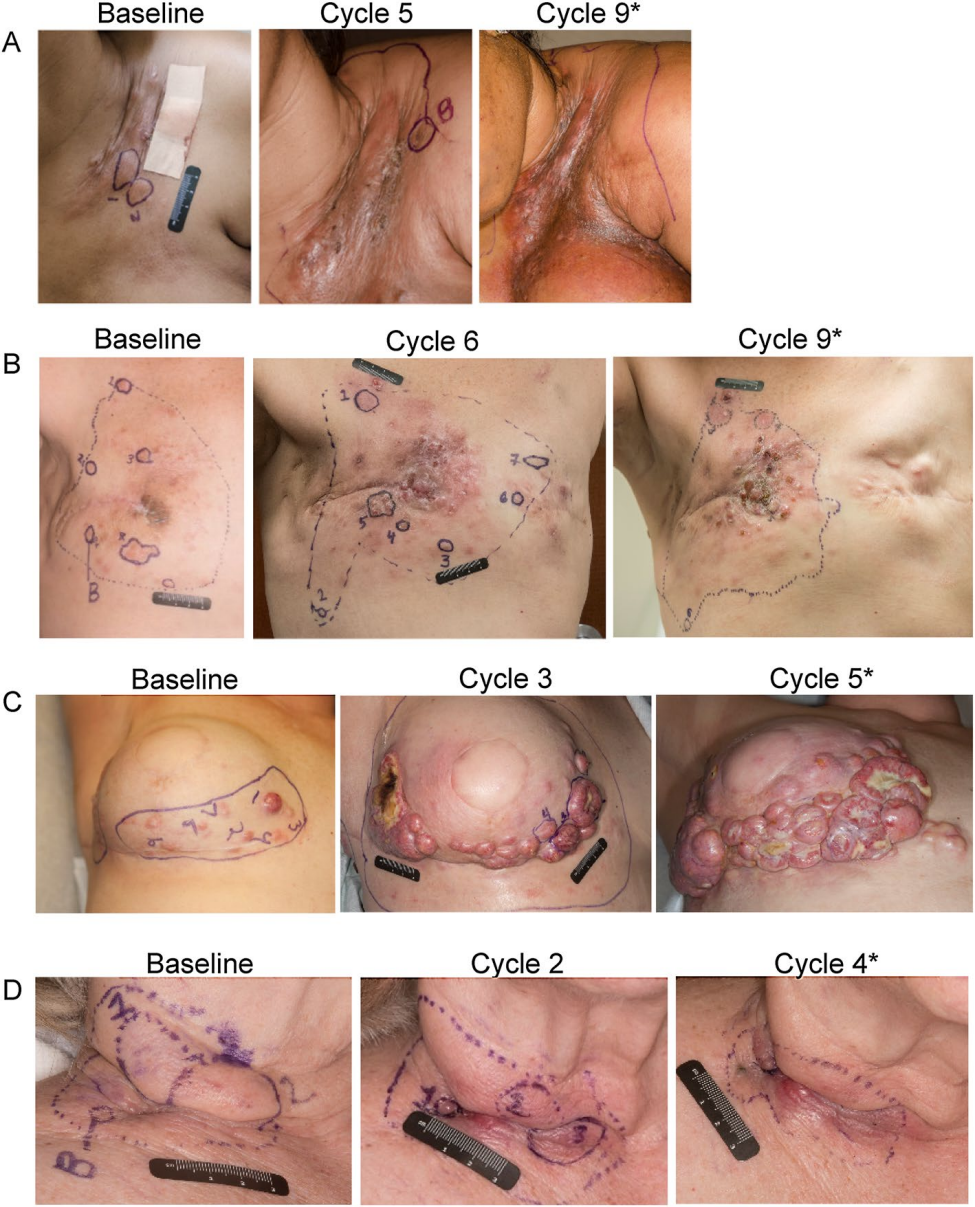
Representative clinical photographs showing the extent of local disease at baseline and before the indicated cycles of T-VEC treatment. Each row corresponds to a single patient. Local reactions after treatment initiation included (**A**) skin scabbing, flattening of lesions, edema, skin thickening, and hyperpigmentation; (**B**) hyperpigmentation, skin thickening, and new lesions; (**C**) increase in tumor burden/new lesions, erythema, and necrosis in tumor; and (**D**) edema, erythema, skin scabbing, and hyperpigmentation. *Patient did not proceed with therapy.

The extent of chest wall/nodal disease at enrollment ranged from a solitary, well-defined lesion to grouped confluent lesions (Fig. 2). After treatment initiation, all patients reported local inflammatory reactions (erythema, edema, pain) at the injection sites, and 3 patients had necrosis in the tumor observed by physical examination. In 5 of the 9 patients, disease at the injection sites was clinically stable for a short period; lesions flattened and scabbed without local progression. The mean duration of this stabilization period was 21.8 days (range, 11–42 days). In the remaining 4 patients, there was no period of disease stabilization. No obvious differences in clinical response or toxicities were observed between HSV-1-seronegative and HSV-1-seropositive patients.

### Toxicity

All 9 patients experienced at least 1 AE that was possibly related to the study drug. The most commonly reported grade 2 or higher AEs were grade 2 injection site reaction, defined as inflammation, erythema, or edema (n = 7); grade 2 injection site pain (n = 5); grade 2 fatigue (n = 4); grade 2 or 3 injection site infection (n = 3); and grade 2 or 3 lymphedema (n = 3) (Table 2). Of the 9 patients, 3 (33%) had a serious AE, defined as an event that necessitated hospitalization for > 24 h. Two patients were hospitalized for injection site infections, and 1 patient was hospitalized for fever.

**Table 2:** Grade 2 or higher adverse events possibly, probably, or definitely related to T-VEC.

Adverse event	Grade 2, n (%)	Grade 3, n (%)
General disorders and injection site conditions		
Fatigue	4 (44)	
Fever	2 (22)	
Injection site reaction	7 (78)	
Tumor/injection site pain	5 (56)	
Gastrointestinal disorders		
Constipation	1 (11)	
Nausea	2 (22)	
Vomiting	2 (22)	
Tumor/injection site infection	1 (11)	2 (22)
Myalgia	1 (11)	
Pain in extremity		1 (11)
Pruritus	1 (11)	
Lymphedema	2 (22)	1 (11)
Neutrophil count decreased	1 (11)	

### Changes in immune phenotype in peripheral blood

**Figure 3:**
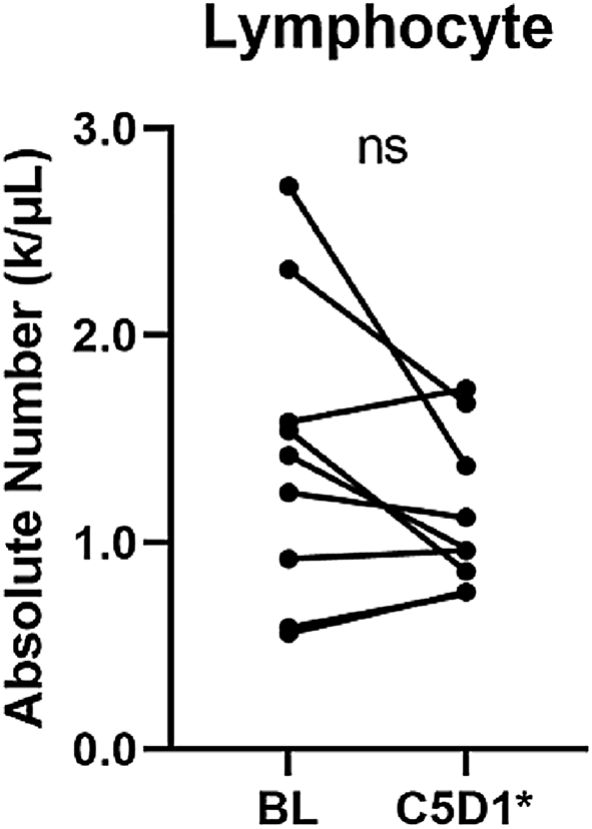
Changes in absolute lymphocyte count. *BL* baseline, *C5D1* Cycle 5 Day 1, *ns* not significant. *One patient's sample was collected before Cycle 4.

**Figure 4:**
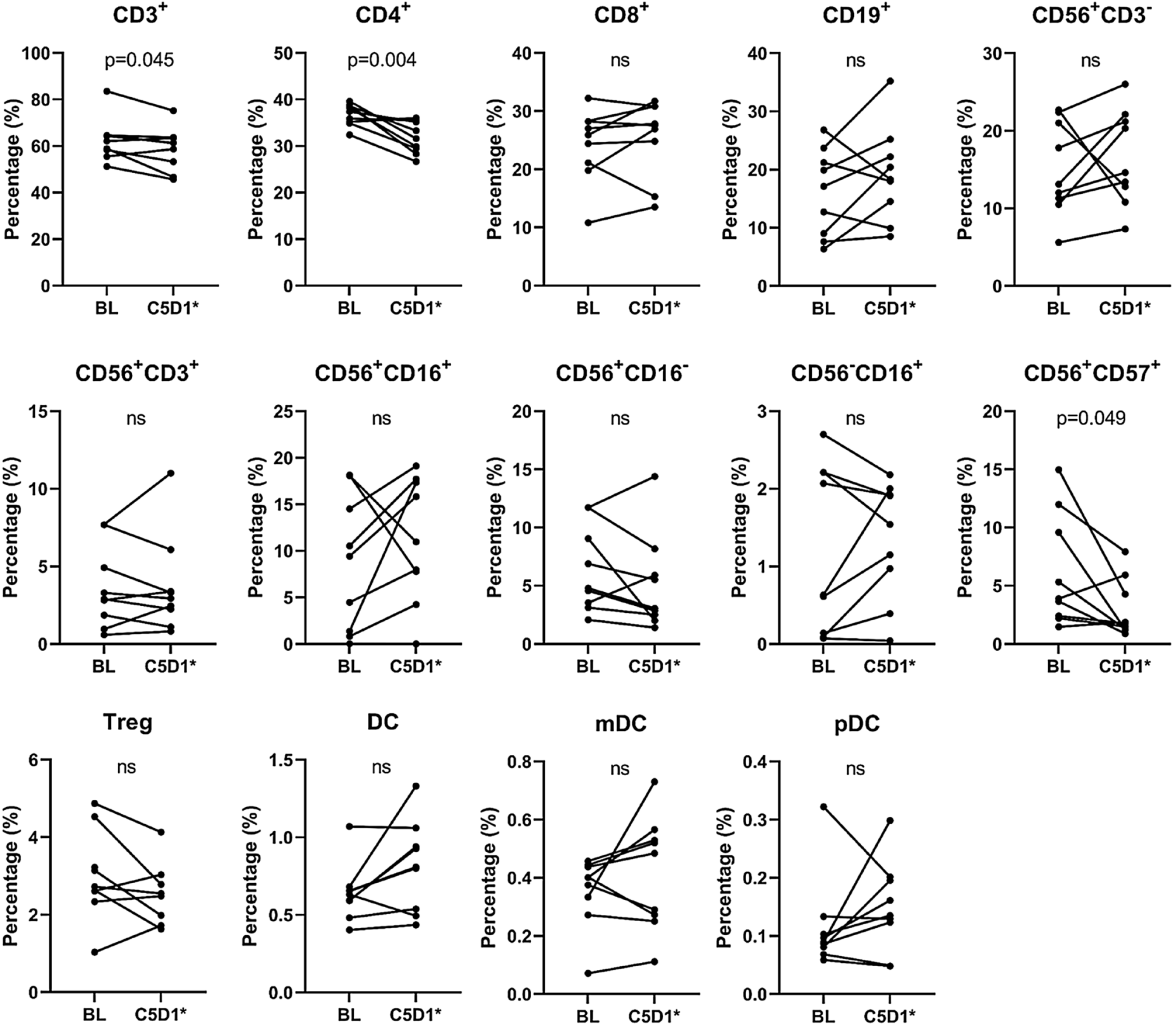
Changes in percentage of immune phenotype. *BL* baseline, *C5D1* Cycle 5 Day 1, *ns* not significant. *One patient's sample was collected before Cycle 4.

There was no significant change in absolute lymphocyte count when we compared baseline and before cycle 5 (Fig. 3). However, the percentage of CD3^+^ and CD4^+^ decreased significantly before cycle 5 compared to baseline (p = 0.045 and p = 0.004, respectively, Fig. 4). Absolute count of CD4 was also significantly decreased (p = 0.027, not shown in Figure). In addition to subsets of T cells (i.e., CD3^+^ or CD4^+^ cells), CD56^+^CD57^+^ NK cells, which are the exhausted NK cells, were also significantly decreased (p = 0.049). Taken together, T-VEC indeed induced a response in a subset of systemic immune cells.

## Discussion

T-VEC was approved by the U.S. Food and Drug Administration for the local treatment of unresectable lesions in patients with recurrent melanoma after initial surgery. This approval was based on the therapeutic benefit demonstrated in a phase III trial in patients with unresectable stage IIIB-IVM1c melanoma, in which T-VEC showed a 31.5% overall response rate with a 16.9% complete response rate^14^. In contrast, in the study we report here, T-VEC monotherapy provided unfavorable disease control in patients with inoperable locoregional recurrence of breast cancer. None of the patients in our study were able to complete 10 cycles of T-VEC as intended per protocol, mainly because of the increase in local tumor burden and/or occurrence of new distant metastases. However, it is worth noting that 1 patient who discontinued the study due to local progression exhibited a great response to concurrent radiotherapy and systemic therapy (immune checkpoint inhibitor and chemotherapy) immediately after this study (not mentioned in “Results”).

T-VEC has the potential to cause tumor lysis, which releases tumor-associated antigens, and GM-CSF, which T-VEC expresses, is a potent immune stimulator that enhances immune responses by activating dendritic cells and T cells to infiltrate the tumor^15,16,19,20^. A previous study showed that tumor-infiltrating lymphocytes in breast cancer are associated with better response to neoadjuvant chemotherapy^21^; thus, intratumoral injection of T-VEC may generate a favorable tumor microenvironment to enhance response to chemotherapy. Indeed, a recent phase I trial of T-VEC combined with neoadjuvant chemotherapy for stage II–III triple-negative breast cancer showed a pathologic complete response rate of 55%, which was higher than the rate of 30–40% expected with neoadjuvant chemotherapy alone^22^. T-VEC was injected intratumorally with intravenous paclitaxel up to 5 times, followed by doxorubicin/cyclophosphamide. Although the sample size was small (9 patients were treated), the results showed increases in cytotoxic T cell (CD8^+^) infiltration in most resected tumor bed specimens along with a reduction in regulatory T cells. These changes in the immune microenvironment of tumors also raised the possibility of further improvement in efficacy with checkpoint inhibitors, attracting significant interest in combination with T-VEC^19,23,24^. Another phase 1b study tested the combination of intrahepatic injection of T-VEC with intravenous atezolizumab in patients with triple-negative breast cancer with liver metastases^25^. No dose-limiting toxicities were observed, and 1 patient had a partial response.

The limitation of our analysis is that assessment of tumor tissues was not possible since none of the patients completed the study. Evaluation of alterations in the immune microenvironment of the injected and non-injected sites, including sites of distant disease, may provide valuable insights regarding the efficacy of T-VEC.

In conclusion, in patients with inoperable locoregional recurrence of breast cancer, intratumoral T-VEC as monotherapy was not therapeutically desirable owing to uncontrolled disease progression. In any future studies of this type of intratumoral immunotherapy for inoperable locoregional breast cancer recurrence, administration of concurrent systemic therapy would be warranted for an optimal outcome.
